# *Chryseobacterium indologenes* in a woman with acute leukemia in Senegal: a case report

**DOI:** 10.1186/1752-1947-8-138

**Published:** 2014-05-06

**Authors:** Arouna Omar, Makhtar Camara, Seynabou Fall, Safietou Ngom-Cisse, Becaye Fall, Awa Ba-Diallo, Halimatou Diop-Ndiaye, Coumba Toure-Kane, Souleymane Mboup, Aissatou Gaye-Diallo

**Affiliations:** 1 Laboratoire de Bactériologie-Virologie, Université Cheikh Anta DIOP, CHU Aristide Le Dantec, BP 7325 Dakar, Senegal; 2 Service de Médecine Interne, Université Cheikh Anta DIOP, CHU Aristide Le Dantec, Dakar, Senegal; 3 Hôpital Principal de Dakar, 1, Avenue Nelson Mandéla, BP 3006 Dakar, Senegal

**Keywords:** *Chryseobacterium indologenes*, Leukemia, Metallo-β-lactamase, Resistance

## Abstract

**Introduction:**

This report documents a rare case of *Chryseobacterium indologenes* urinary tract infection in Senegal. *Chryseobacterium indologenes* is an uncommon human pathogen reported in hospital outbreaks in Taiwan and there have been some sporadic cases reported in Europe and in the USA mainly from immune-suppressed patients.

**Case presentation:**

This case report describes a 42-year-old woman of Wolof^a^ ethnicity who was hospitalized in our Department of Internal Medicine in a Senegalese university teaching hospital, with acute leukemia who died of severe sepsis 10 days following her hospitalization. A strain of *Chryseobacterium indologenes* isolated from her urine sample was resistant to several beta-lactams including ampicillin (minimum inhibitory concentrations ≥256μg/mL), cefotaxime (minimum inhibitory concentrations 32μg/mL) and imipenem (minimum inhibitory concentrations ≥32μg/mL), whereas it was susceptible to piperacillin (minimum inhibitory concentrations 16μg/mL), cefepime (minimum inhibitory concentrations 4μg/mL), ceftazidime (minimum inhibitory concentrations 4μg/mL), trimethoprim-sulfamethoxazole (minimum inhibitory concentrations ≤0.25μg/mL) and all tested quinolones including nalidixic acid (minimum inhibitory concentrations ≤2μg/mL).

**Conclusions:**

*Chryseobacterium indologenes* although uncommon, is an important pathogen causing infection in hospitalized patients. The management of this infection needs better identification, drug susceptibility testing and monitoring of immunosuppressed patients with long hospitalizations.

## Introduction

*Chryseobacteria* are a group of Gram-negative, non-fermenting, non-motile, catalase-, oxidase- and indole-positive aerobic bacilli. *Chryseobacterium indologenes* was first isolated from the tracheal aspirate of a patient with ventilator-associated pneumonia in 1993. *Chryseobacterium* species rarely cause human infections [1] but has been reported in nosocomial infections in Taiwan and rarely elsewhere [2]. In the hospital environment, *C. indologenes* is found in water systems, sink basins, the surfaces of equipment and wet medical devices (such as ventilators, humidifiers, and suction tubes) [3,4]. Despite their low virulence, *Chryseobacteria* are inherently resistant to many antimicrobial agents including imipenem.

*C. indologenes* from urinary tract infection was reported in Burkina Faso, India and Spain [5-7]. In Senegal, two cases of meningitis due to *Flavobacterium meningosepticum* were diagnosed in the late-1970s [8], but *C. indologenes* has not been reported previously.

## Case presentation

A 42-year-old woman of Wolof^a^ ethnicity, who underwent eight pregnancies (of which one was aborted), with chronic myeloid leukemia (CML), was admitted to our Intensive Care Unit (ICU) 1 year before this report for a tonic–clonic generalized seizure 11 days after a normal vaginal delivery (sixth child). At this date, a clinical examination showed a blood pressure of 130/70 (mmHg), a fever of 37.6°C and a tachycardia of 125 beats per minute. Her Glasgow Coma Score (GCS) was 13/15 (E4M5V4) with reactive pupils and without localizing signs; her reflexes were present and normal. For biological parameters, no albumin was found in her urine or at blood level; hypernatremia of 148mEq/L, hypokalemia 2.4mEq/L, anemia 11.6g/dL, thrombocytosis of 729,000/mm^3^ and a leukocytosis of 123,000 white blood cells (WBC)/mL (lineage not given). After symptomatic treatment including rehydration, her GCS returned to 15/15 and her serum electrolytes normalized, she was then referred to hematology for further investigation.

The diagnosis of CML was confirmed by a myelogram and a finding of Bcr-Abl fusion positive genotype (Philadelphia chromosome) a year prior to her ICU hospitalization. She was then treated with imatinib (mesylate) 100mg (Glivec®) under a standard protocol of two tablets two times a day. This treatment continued until the beginning of her subsequent, seventh pregnancy at an unknown time.

Two months after giving birth (eighth pregnancy), she was hospitalized again (day 0), with hepatomegaly, splenomegaly type IV according to Hackett’s classification, an anemic syndrome and an infectious syndrome with a temperature of 38.8°C. Her blood count showed leukocytosis of 275,000 WBC/mL cells with 40% of blast cells, therefore an acute crisis of her CML was suspected. An abdominal ultrasound confirmed a homogeneous hepatosplenomegaly without signs of portal hypertension. Blood culture and urine culture were requested, but not performed due to lack of finance. Glivec® (imatinib) was given at a dose of 600mg per day. Empirical antibiotic therapy of ceftriaxone 2g daily was administered. It is only on day 7 that a urine sample was taken for cytology and bacteriology examination at a laboratory. She did not recover, by day 8 she was in septic shock and she subsequently died on day 10 with severe sepsis.

**Figure 1:**
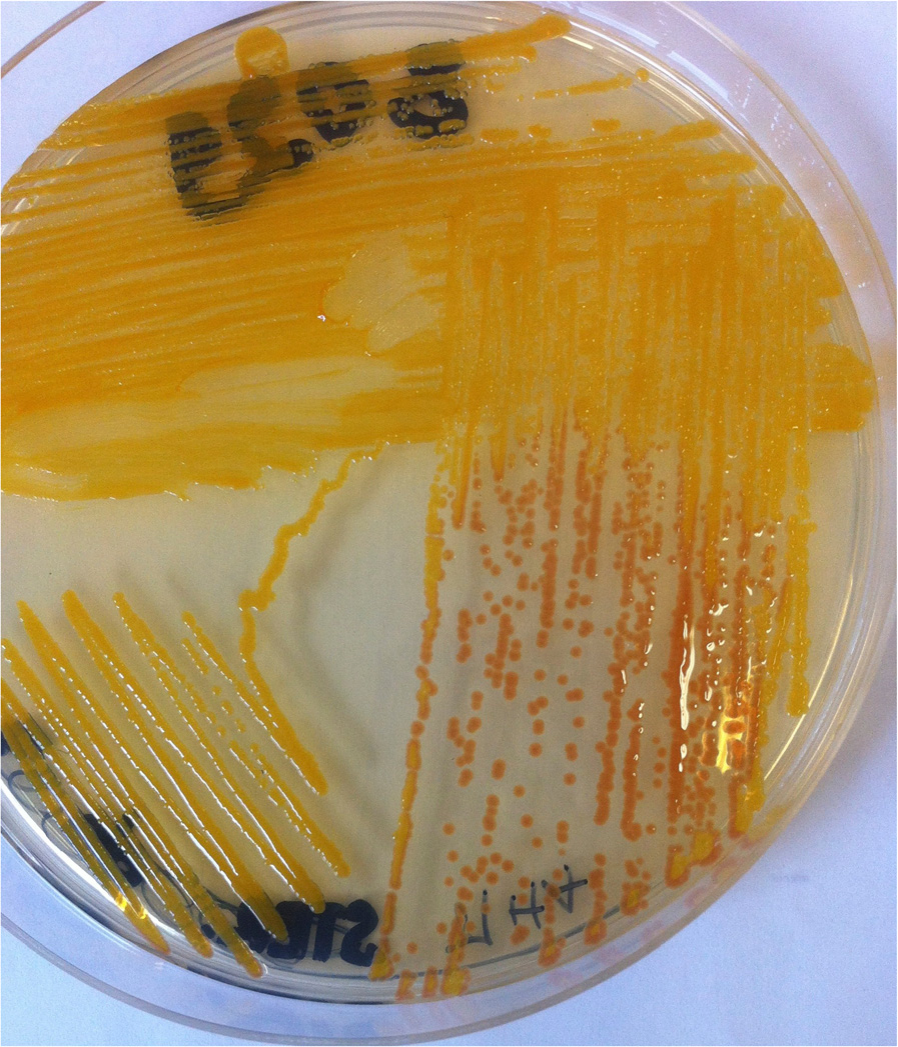
Yellow-pigmented colonies changed from yellow to red by adding 1 drop of 10% sodium hydroxide (flexirubin).

The urine received from the day before her death was clear with a rich bacterial flora but few cells in cytology. Microscopy showed Gram-negative bacilli. Her urine was inoculated on cysteine lactose electrolyte deficient (CLED) agar in accordance with the usual techniques of medical bacteriology. The CLED agar grew yellow-colored, 1 to 2mm circular colonies (>10^7^ CFU/mL) with regular margins. Similar yellow-pigmented colonies were also observed on Müller-Hinton Agar (Figure 1). The flexirubin type of pigment was confirmed by adding 1 drop of 10% sodium hydroxide solution to a bit of cell paste. The color of the colonies changed from yellow to red (Figure 1).

**Figure 2:**
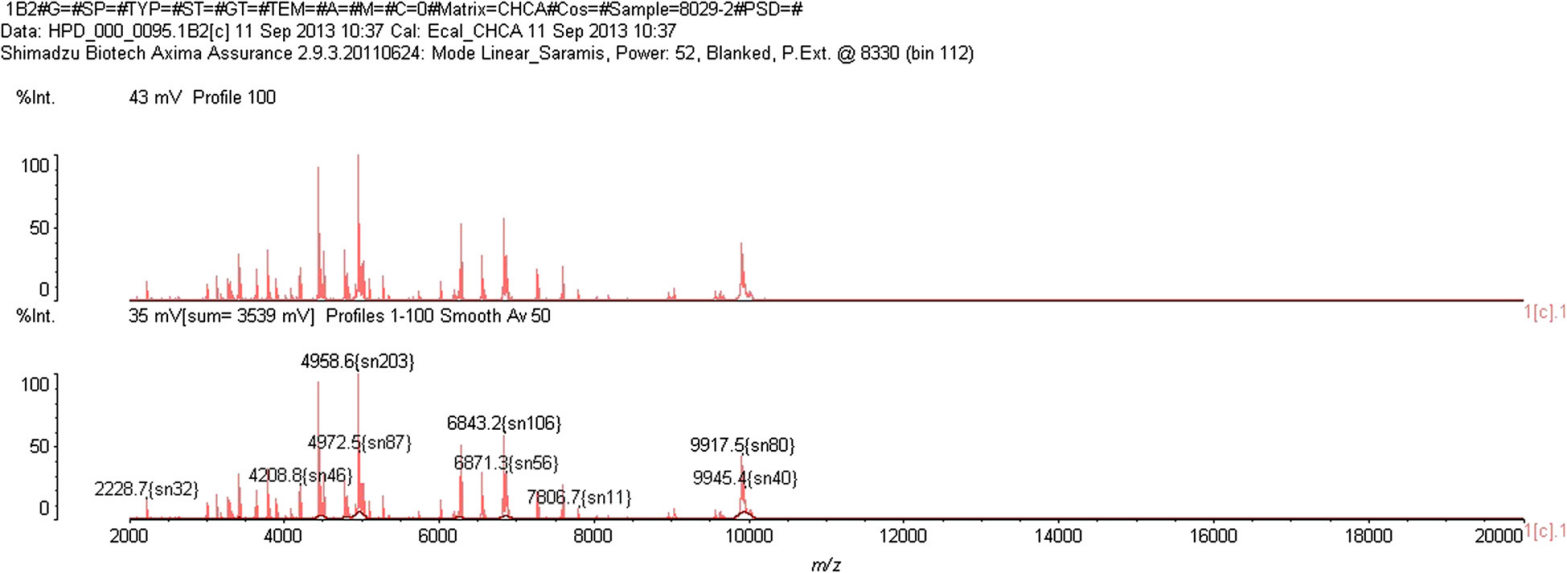
Mass spectral profile.

**Figure 3:**
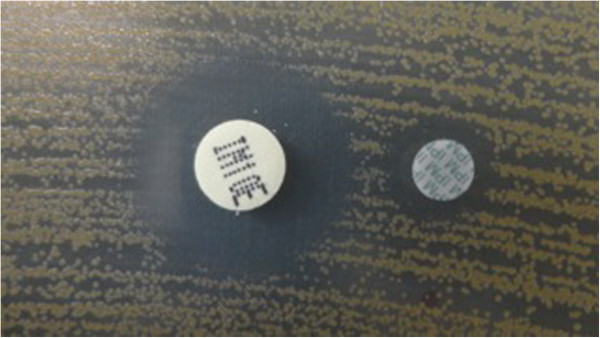
Evidence of metallo-β-lactamase producing resistance to imipenem with double-disc synergy test (ethylenediaminetetraacetic acid and imipenem).

After culture, *C. indologenes* was suspected by microscopy examination and biochemical tests (Gram-negative bacilli that were non-motile, produced oxidase, catalase, and urease and indole). *C. indologenes* was confirmed using the Appareil et Procédés d’Identification 20NE identification system (bioMérieux, France; excellent identification % Id = 99.9 and T = 0.82). The genus *Chryseobacterium* was confirmed by mass spectrometry (Vitek MS matrix-assisted laser desorption/ionization, time-of-flight, bioMérieux) in an army teaching hospital (Figure 2). Antimicrobial susceptibility testing by disc diffusion and dilution on microplate for minimal inhibition concentration methods using *Pseudomonas aeruginosa* ATCC 27853 and *Escherichia coli* ATCC 25922 for internal quality control was done and interpreted according to the Comité d’Antibiogramme de la Société Française de Microbiologie recommendations of 2013. This strain was susceptible to piperacillin, ceftazidime, trimethoprim-sulfamethoxazole, and quinolone including nalidixic acid and resistant to cefotaxime, aztreonam, imipenem, gentamicin, amikacin, tobramycin, and colistin (Table 1). A test for metallo-β-lactamase (MBL) was done and the result was positive (Figure 3). Hydrolysis tests performed with imipenem (substrate) and a crude extract of a liquid medium culture of our strain, the objective being the production of beta-lactamase, by measuring the absorbance variation with a Cary 100 UV-visible spectrophotometer (Varian, Walnut Creek, CA, USA). Imipenem hydrolysis activity inhibited (>90%) after incubation with 5mM ethylenediaminetetraacetic acid, revealed the presence of an MBL. To characterize the gene encoding MBL of the strain, polymerase chain reaction (PCR) was carried out using genomic deoxyribonucleic acid (DNA) obtained by phenol–chloroform extraction, as the template primers IND-INV/+ (5′-TTGGCAGAATATTCTTTACC) and IND-INV/– (5′-GAAAAAAAGACGGAAAAGCAAC) as described previously by Bellais and coworkers [9].

**Table 1:** ***In vitro *** **antimicrobial susceptibilities of the strain, and interpretation according to Comité d’Antibiogramme de la Société Française de Microbiologie 2013 recommendations, using *Pseudomonas aeruginosa *** **ATCC 27853 and *Escherichia coli *** **ATCC 25922 as quality control**

**Antibiotics**	**MIC (μg/mL)**	**IZD (mm)**	**Breakpoint (S)**		**Categorization**
			**μg/mL**	**mm**	
Ampicillin	≥256	0	≤16	≥22	Resistant
Piperacillin	16	20	≤16	≥18	Susceptible
Ticarcillin	≥256	0	≤16	≥22	Resistant
Temocillin	≥256	0	≤16	≥22	Resistant
Cephalothin	≥256	0	≤16	≥22	Resistant
Cefuroxime	≥256	0	≤16	≥22	Resistant
Cefotaxime	32	8	≤16	≥22	Resistant
Ceftazidime	4	25	≤08	≥19	Susceptible
Ceftriaxone	32	8	≤16	≥22	Resistant
Cefepime	4	28	≤08	≥19	Susceptible
Cefotetan	≥32	6	≤16	≥22	Resistant
Imipenem	≥32	6	≤04	≥22	Resistant
Meropenem	32	6	≤02	≥22	Resistant
Ertapenem	≥32	6	≤02	≥22	Resistant
Aztreonam	≥32	8	≤01	≥27	Resistant
Trimethoprim-sulfamethoxazole	≤0.25	22	≤2	≥16	Susceptible
Nalidixic acid	≤2	28	≤08	≥20	Susceptible
Gentamicin	ND	4	ND	≥16	Resistant
Amikacin	ND	0	ND	≥17	Resistant
Tobramycin	ND	0	ND	≥16	Resistant
Colistin	ND	0	ND	–	Resistant

Abbreviations: IZD, inhibition zone diameter; MIC, minimum inhibitory concentration. ‘ND’, Not Determined; ‘–’ Colistin breakpoint not given. S, sensitivity.

## Discussion

*Chryseobacterium* is an uncommon pathogen reported to have caused hospital-acquired infection in Taiwan. Six cases have been reported in Europe, two in Australia, two in India and seven in the USA [6]. To the best of our knowledge, this case report of *C. indologenes* infection is the second in Africa, the first was described in Burkina Faso in 2009 [5].

The pathogenicity of *C. indologenes* is not well established, however, it is already known that biofilm and proteases production are important mechanisms involved in its virulence [10]. *Chryseobacterium* is often encountered in water, soil and plants. Resistant to chlorination, it is found in the hospital environment at water systems and on medical equipment. Colonization of patients via contaminated medical devices involving fluids such as respirators, intubation tubes, humidifiers, and incubators for newborns has been reported [10]. The rehabilitation of our Department of Internal Medicine just after the death of our patient, has not allowed us to take environmental samples to investigate the source of infection.

The associated infections involve the blood stream, pneumonia, intra-abdominal and urinary tract [6,7,11] and the main risk factors are oncological disease, long hospital stays and prolonged antibiotic treatment (>14 days) [10-12]. Our patient had CML, which evolved into an acute leukemia and she was hospitalized with infectious syndrome. A blood culture was requested but it was not done due to lack of financial resources. Clinical data allow us to state that she died of septicemia; however, the isolation of the bacterium only in her urine does not allow us to confirm that it is responsible, although urinary tract infection criteria are met. Nevertheless, it is possible that the bacterium was responsible for septicemia due to her immunocompromised state; several cases of bacteremia due to *C. indologenes* have been published [11]. The majority of the cases reported in the literature with infections caused by *C. indologenes* had critical diseases and, frequently, polymicrobial infections.

Only 0.03% (50 of 155,811) of all bacterial isolates collected by the SENTRY Antimicrobial Surveillance Program [13] during the 5-year period 1997 to 2001 were members of the genera *Chryseobacterium* or *Elizabethkingia* (formerly *Chryseobacterium*), the most frequent organisms being *Elizabethkingia meningoseptica* (formerly *Chryseobacterium meningosepticum*), *C. indologenes* and *Chryseobacterium gleum*[14]*.* All 50 isolates were from hospitalized patients, and the vast majority was recovered from either the lower respiratory tract (52.0%) or blood cultures (46.0%). Among the isolates from bloodstream infections, 30.4% were *C. indologenes*.

According to the results of the SENTRY Antimicrobial Surveillance Program, the antimicrobials most active against *C. indologenes* are the quinolones and trimethoprim-sulfamethoxazole (≥95% susceptibility), followed by piperacillin-tazobactam (90% susceptibility). Ciprofloxacin, cefepime, ceftazidime, piperacillin, and rifampin showed reasonable activity (85% susceptibility), whereas aminoglycosides, other β-lactams, chloramphenicol, linezolid, and glycopeptides are not appropriate for treating infections by this organism [13]. The strain was found to be resistant to imipenem (minimum inhibitory concentrations, MICs, ≥32μg/mL) due to MBL (class B) production and susceptible to piperacillin (MICs 16μg/mL), cefepime (MICs 4μg/mL), ceftazidime (MICs 4μg/mL) trimethoprim-sulfamethoxazole (MICs ≤0.25μg/mL) and all quinolones including nalidixic acid (MICs ≤2μg/mL). It is also resistant to ceftriaxone (MICs 32μg/mL), used as empirical antibiotic therapy for our patient. Our strain seemed to be less resistant than *C. indologenes* 597 (IND-6) of Burkina Faso according to MIC of imipenem (≥32μg/mL versus ≥64μg/mL) and quite more resistant than *C. indologenes* NF 16 (CDC group IIb, IND-5) (≥32μg/mL versus 32μg/mL). Several species of *Flavobacteriaceae*, including *C. indologenes,* are naturally resistant to β-lactam antibiotics (including carbapenems) due to production of a resident MBL (class B) IND-1 for indologenes [9]. Seven variants of MBL (class B; IND-1 to IND-6 and IND-2a) have been detected in *C. indologenes.* A new IND-type variant named IND-7 has also been recently reported from Japan (2010) in a *C. indologenes* isolate [15]. Nonspecific bands obtained by PCR-IND for our strain can be explained by the sequence heterogeneity at the 5′ and 3′ extremities of the MBL gene, which made unsuccessful direct amplification with consensus primers designed to amplify all known IND-like MBL genes. An inverse PCR approach to obtain the complete sequence of the *bla*_*IND*_ of our strain is in progress.

## Conclusions

*C. indologenes* although uncommon, is an important pathogen causing infection in hospitalized patients. The management of this infection needs better identification, susceptibility testing and monitoring of immunocompromised patients with long hospital stays. In addition, this report showed the necessity to survey environmental bacteria that could cause hospital-acquired infection.

## Consent

The signed written consent of the husband of the deceased patient was obtained for publication of this case report and the accompanying images after explaining the interest of this publication. A copy of the written consent is available for review by the Editor-in-Chief of this journal.

## Endnote

^a^An ethnic group in Senegal, West Africa.

## Competing interests

All authors declare that they have no competing interests.

## Authors’ contributions

AO, CM, FS, NCS, FB, BDA, DNH, TKC, MS and GDA conducted the case review and contributed to the writing of the case report. All authors read and approved the final report.
